# Soybean *Gm*SAUL1, a Bona Fide U-Box E3 Ligase, Negatively Regulates Immunity Likely through Repressing the Activation of *Gm*MPK3

**DOI:** 10.3390/ijms24076240

**Published:** 2023-03-25

**Authors:** Jun-Mei Li, Mei-Yan Ye, Chaofeng Wang, Xiao-Han Ma, Ni-Ni Wu, Chen-Li Zhong, Yanjun Zhang, Ninghui Cheng, Paul A. Nakata, Lirong Zeng, Jian-Zhong Liu

**Affiliations:** 1 Institute of Plant Genetics and Developmental Biology, College of Chemistry and Life Sciences, Zhejiang Normal University, Jinhua 321004, China; 2 Zhejiang Provincial Key Laboratory of Biotechnology on Specialty Economic Plants, Zhejiang Normal University, Jinhua 321004, China; 3 Center for Plant Science Innovation, University of Nebraska-Lincoln, Lincoln, NE 68588-0666, USA; 4 U.S. Department of Agriculture-Agricultural Research Service, Children’s Nutrition Research Center, Department of Pediatrics, Baylor College of Medicine, Houston, TX 77030, USA

**Keywords:** *Glycine max*, E3 ligase, *GmSAUL1*, virus-induced gene silencing, immune responses, cell death, soybean

## Abstract

E3 ubiquitin ligases play important roles in plant immunity, but their role in soybean has not been investigated previously. Here, we used *Bean pod mottle virus* (BPMV)-mediated virus-induced gene silencing (VIGS) to investigate the function of *Gm*SAUL1 (Senescence-Associated E3 Ubiquitin Ligase 1) homologs in soybean. When two closely related *SAUL1* homologs were silenced simultaneously, the soybean plants displayed autoimmune phenotypes, which were significantly alleviated by high temperature, suggesting that *Gm*SAUL1a/1b might be guarded by an R protein. Interestingly, silencing *GmSAUL1a/1b* resulted in the decreased activation of *Gm*MPK6, but increased activation of *Gm*MPK3 in response to flg22, suggesting that the activation of *Gm*MPK3 is most likely responsible for the activated immunity observed in the *Gm*SAUL1a/1b-silenced plants. Furthermore, we provided evidence that *Gm*SAUL1a is a bona fide E3 ligase. Collectively, our results indicated that *Gm*SAUL1 plays a negative role in regulating cell death and immunity in soybean.

## 1. Introduction

Plants ward off numerous pathogens through multi-layer defenses, including nonhost resistance, pathogen-associated molecular patterns (PAMP)-triggered immunity (PTI) or basal resistance, and effector-triggered immunity (ETI) [1,2]. The detection of PAMPs by plasma-membrane-localized pattern recognition receptors (PPRs) activates PTI [3,4], whereas the specific recognition of pathogen-delivered effectors by R proteins or NLRs (nucleotide-binding leucine-rich repeat proteins) leads to ETI [2]. Although divergent signaling exists between PTI and ETI [5], they activate a similar set of overlapping responses including the induction of *pathogenesis-related* (*PR*) genes, the activation of mitogen-activated protein kinases (MAPKs) and calcium-dependent protein kinases, and the hypersensitive response (HR) cell death [6,7]. However, the activation of ETI is higher in magnitude and lasts longer than PTI [2].

Ubiquitination is a common post-translational modification in eukaryotes for the regulation of the stability of protein substrates [8,9]. Ubiquitin and ubiquitin-like modifiers have evolved significantly to fulfill diverse functions in plants. Poly-ubiquitinated protein substrates linked via the Lys^48^ residue of ubiquitin is marked and subsequently targeted for degradation by the 26S proteasome, whereas Lys^63^-linked ubiquitination usually plays non-proteolytic regulatory roles. The ubiquitination reaction consists of three steps catalyzed sequentially by a ubiquitin-activating enzyme (E1), a ubiquitin-conjugating enzyme (E2), and a ubiquitin ligase (E3) [8,10]. Among these three enzymes, E3 ligases are the key enzymes that determine substrate specificity [11]. The Arabidopsis genome consists over 1400 genes that encode E3 ligase-related proteins [12], implying their diverse functions in numerous biological processes. E3 ligases are classified into different families: HECT, SCF, RING-finger, U-box, and APC [13,14]. Among these E3-ligase families, F-box proteins are involved in the regulation of hormone signaling [15], whereas U-box E3 ligases have been reported playing critical roles in plant immunity [13,14]. Rice *SPL11* encodes a protein with a central U-box domain and carboxyl-terminal armadillo (ARM) repeat domain and the *spl11* mutant displays an autoimmune response with enhanced disease resistance [16]. The Arabidopsis U-box (PUB) proteins PUB12 and PUB13 negatively regulate PTI responses through targeting the flagellin receptor FLS2 (Flagellin-Sensitive 2) for degradation [17]. A triplet of U-box protein paralogs, PUB22, PUB23, and PUB24, are involved in PTI through redundantly targeting the exocyst complex component EXO70B2 for degradation [18].

Arabidopsis senescence-associated E3 ubiquitin ligase 1 (SAUL1) was initially identified as a U-box E3 ubiquitin ligase required for the suppression of premature senescence [19]. Recently, it has been shown that a loss-of-function mutation in SAUL1 results in enhanced disease resistance against multiple biotrophic pathogens [20,21,22,23,24]. This autoimmunity is totally dependent on Enhanced Disease Susceptibility 1 (EDS1), Phytoalexin Deficient 4 (PAD4), and Suppressor of the G2 Allele of SKP1b (SGT1B) [20,21,22]. From *saul1-1* suppressor screening, the TIR-type NLR (TNL) protein SUSA1 (Suppressor of *saul1-1*), previously referred to as SOC3 (Suppressor of *chs1-2*, 3) [25], was identified as a guard for SAUL1 [23]. Interestingly, the autoimmunity triggered by the overexpression of *SAUL1* also depends on SOC3 [23]. These results indicate that the homeostasis of SAUL1 is monitored by SOC3. A level of SAUL1 exceeding a certain threshold range will lead to the activation of immunity. Liang et al. recently uncovered SOC3 pairs with different truncated TIR-NB (TN) proteins to monitor either the absence or overexpression of SAUL1, respectively [24].

Mitogen-activated protein kinase (MAPK) cascades are major components downstream of PTI and ETI to transduce defense signals [26,27,28]. Increasing evidence indicates that MPK3 and MPK6 play positive roles in immunity [26,28,29,30,31,32,33], whereas MPK4 plays both positive and negative roles in regulating plant immunity [34,35,36,37,38]. Using virus-induced gene silencing (VIGS) mediated by *Bean pod mottle virus* (BPMV), we demonstrated that, while soybean *Gm*MPK4 plays a negative role, *Gm*MPK6 plays both positive and negative roles in defense responses [39,40].

The BPMV-VIGS system is one of the most successful tools in functional genomics studies in paleoploidy soybean [41,42], in which most of the genes in its genome have two copies [43]. In this study, we sought to identify the E3 ligases with critical roles in soybean immunity by reverse genetic screening using the BPMV-VIGS system. We found that when two closely related *SAUL1* homologs, *GmSAUL1a* and *GmSAUL1b*, were silenced simultaneously, the soybean plants exhibited autoimmune phenotypes with enhanced resistance to different biotrophic viral and bacterial pathogens, indicating that the *Gm*SAUL1a/1b plays a negative role in immune responses in soybean. Treatment of the *GmSAUL1a/1b*-silenced plants at 30 °C significantly alleviated the auto-immune phenotypes, suggesting that *Gm*SAUL1a/1b might be guarded by an R protein. Interestingly, we found that silencing the *GmSAUL1a/1b* in soybean resulted in the differential activation or repression of downstream MPK3 and MPK6. Collectively, our results indicated that *Gm*SAUL1a/1b plays a negative role in regulating cell death and immunity.

## 2. Results

### 2.1. Silencing GmSAUL1 Results in a Constitutively Activated Immune Responses in Soybean

**Figure 1:**
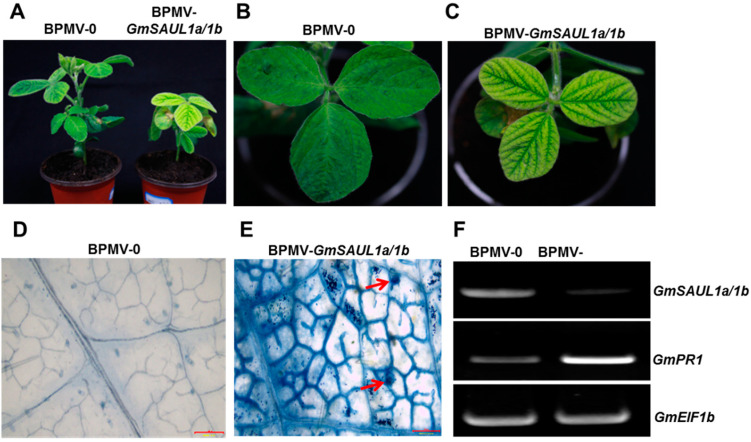
Silencing *GmSAUL1a/1b* results in activated immune responses in soybean. (**A**) Silencing *GmSAUL1a/1b* led to stunted stature in soybean; comparison of the leaves of the BPMV-0 plants (**B**) and the leaves of the BPMV-*GmSAUL1a/1b* plants (**C**). Cell death was observed on the leaves of the BPMV-*GmSAUL1a/1b* plants, but not on the leaves of the BPMV-0 plants. (**D**) BPMV-0 leaf stained with Trypan blue. (**E**) BPMV-*GmSAUL1a/1b* leaf stained with Trypan blue. The dead cells are pointed out by the red arrows. (**F**) RT-PCR analysis showing that the *GmSAUL1a/1b* transcript was significantly silenced in the *GmSAUL1a/1b*-silenced plants relative to the BPMV-0 plants and the expression of the *GmPR1* was significantly induced in the *GmSAUL1a/1b*-silenced plants relative to the BPMV-0 plants. Bars = 0.5 mm.

**Figure 2:**
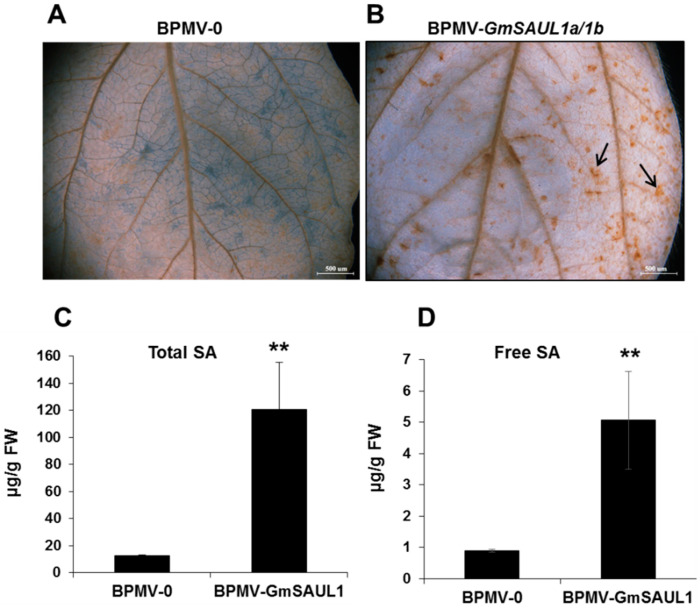
Silencing *GmSAUL1a/1b* results in the increased accumulation of both H_2_O_2_ and SA. (**A**) Presence of H_2_O_2_ on the leaves of BPMV-0 plants by DAB staining. (**B**) Presence of H_2_O_2_ on the leaves of the *GmSAUL1a/1b*-silenced plants. Oxidized DAB formed a reddish-brown deposit (examples of these deposits are indicated by the black arrows). Both total SA levels (**C**) and free SA (**D**) were quantified in *GmSAUL1a/1b*-silenced and BPMV-0 empty vector control plants at 20 days post BPMV inoculation. Error bars represent SD for three independent samples. Double asterisks indicate significant differences from the control (**, *p* < 0.01, Student’s *t* test). FW, fresh weight.

To identify the genes involved in immunity in soybean, we performed reverse genetic screening using the BPMV-VIGS system. When two U-box genes were silenced simultaneously, the soybean plants exhibited an auto-immune phenotype, including stunted stature (Figure 1A), cell death on the leaves (compare Figure 1B with Figure 1C and Figure 1D with Figure 1E), induced expression of *PR1* (Figure 1F, middle gel)*,* and the over-accumulation of both H_2_O_2_ (compare Figure 2A with Figure 2B) and salicylic acid (SA) (Figure 2C,D). The high levels of H_2_O_2_ and SA could be the primary reasons for the auto-immune phenotype observed on the silenced plants. Blast searching using the 360 bp fragment inserted in the BPMV-2 vector against the soybean genome (Phytozome 12) revealed that there are two paralogous genes in the soybean genome that share the highest homology (~80%) with the Arabidopsis SAUL1 (senescence-associated E3 ubiquitin ligase, also known as PUB44/At1G20780). Therefore, we referred to these two genes as *GmSAUL1a* (Glyma.04G016500) and *GmSAUL1b* (Glyma.06G016500), respectively. *GmSAUL1a* and *GmSAUL1b* share 98.6% identity at the nucleotide level. As the VIGS approach can simultaneously silence genes sharing 85% identity at the nucleotide level [39,44,45,46,47], we believe that the auto-immune phenotype was a result of silencing both *GmSAUL1a* and *GmSAUL1b* (Figure 1F, upper gel). These results proved again that VIGS is a robust tool in gene function studies in paleotetraploidy soybean plants [41,42].

### 2.2. Silencing GmSAUL1a/1b Leads to Enhanced Resistance to Biotrophic Bacterial and Viral Pathogens

**Figure 3:**
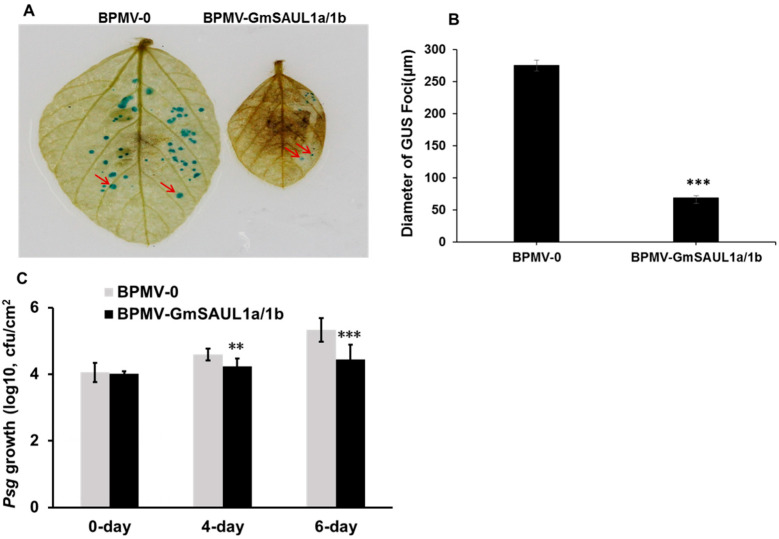
Silencing *GmSAUL1a/1b* enhances the resistance of soybean plants to both SMV and *Pseudomonas syringae pv*. *glycinea* (*Psg*). (**A**) Enhanced resistance of the *GmSAUL1a/1b-*silenced plants to SMV-N-GUS. At 3 weeks post inoculation of the soybean plants with BPMV-0 or BPMV-*GmSAUL1a/1b*, the SMV-N-GUS was bombarded into the detached leaves of silenced and non-silenced plants. At 5 days post inoculation (dpi) with SMV-N-GUS, the presence of SMV-N-GUS in the infected leaves was detected by GUS staining. Red arrows pointed to the representative GUS foci. (**B**) Comparison of the diameters of SMV-N-GUS foci on the leaves of BPMV-0 and BPMV-*GmSAUL1a/1b* plants. Error bars represent SD of the diameters of at least 60 GUS foci measured on each of three independent leaves. Asterisks indicate a significant difference from the control (***, *p* < 0.001, Student’s *t* test). (**C**) *Psg* growth curves on the leaves of vector control plants and *GmSAUL1a/1b*-silenced plants at different days post inoculation (dpi). ** indicates significant difference at 0.01 level by Student’s *t* test. *** indicates significant difference at 0.001 level by Student’s *t* test.

The auto-immune phenotype is usually associated with enhanced resistance. To examine whether the *GmSAUL1a/1b*-silenced plants exhibit enhanced resistance to biotrophic pathogens, we performed disease resistance assays on both the vector control plants (BPMV-0) and the *GmSAUL1a/1b*-silenced plants. Firstly, we inoculated three individual leaves detached from both the BPMV-0 plants and *GmSAUL1a/1b*-silenced plants, respectively, with a SMV (*soybean mosaic virus*) strain tagged with the GUS (β-glucuronidase) protein (SMV-N-GUS) [48] via biolistic bombardment. At 5 days post inoculation (dpi), the SMV-N-GUS infection was visualized by GUS staining. As shown in Figure 3A,B, the GUS foci on the *GmSAUL1a/1b*-silenced plants were much smaller than on the BPMV-0 plants, indicating that *Gm*SAUL1a/1b plays a negative role in SMV resistance.

To examine the effects of *GmSAUL1* silencing on the resistance of soybean against bacterial pathogens, the *Pseudomonas syringae pv. glycinea* (*Psg*) R4 strain was inoculated by directly spraying bacterial solutions on the leaves of both the BPMV-0 and the *GmSAUL1a/1b*-silenced plants, respectively. As shown in Figure 3C, the multiplication of *Psg* was significantly higher on the BPMV-0 leaves than on the leaves of the *GmSAUL1a/1b*-silenced plants. Together, these results indicated that silencing *GmSAUL1a/1b* enhances the resistance of soybean plants to both viral and bacterial pathogens.

### 2.3. Autoimmune Phenotype of GmSAUL1a/1b-Silenced Plants Is Significantly Suppressed by Higher Temperature Treatment

**Figure 4:**
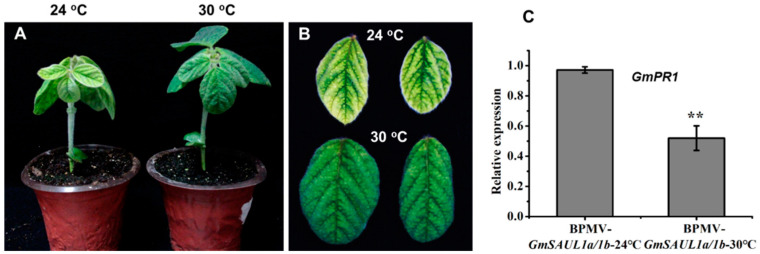
High temperature (30 °C) could partially suppress the auto-immune phenotype of the *GmSAUL1a/1b*-silenced plants. Comparison of the whole plant phenotype (**A**) and the leaf phenotype (**B**) of the *GmSAU1a/1b*-silenced plants grown at 24 °C and 30 °C. (**C**) *GmPR1* gene expression of the *GmSAUL1a/1b*-silenced plants is significantly reduced at 30 °C relative to at 24 °C. ** indicate significant level at 0.01%.

Autoimmunity resulting from *R* gene activation is usually suppressed at high temperatures [49,50,51]. To further examine whether the autoimmunity of the *GmSAUL1a/1b*-silenced plants is a result of NLR activation, the *GmSAUL1a/1b*-silenced plants were subjected to treatment at 30 °C. As expected, the autoimmune phenotype of the *GmSAUL1a/1b*-silenced plants was significantly alleviated at a higher temperature (30 °C) (Figure 4). Accordingly, the induction of *PR1* expression was also significantly lower at 30 °C than at 24 °C (Figure 4C), confirming that the autoimmunity observed in the *GmSAUL1a/1b*-silenced plants is likely to be a result of the activation of NLR protein(s).

### 2.4. Silencing GmSAUL1a/1b Exhibits Opposite Effects on the Activation of GmMPK3 and GmMPK6 in Response to flg22 Treatment

**Figure 5:**
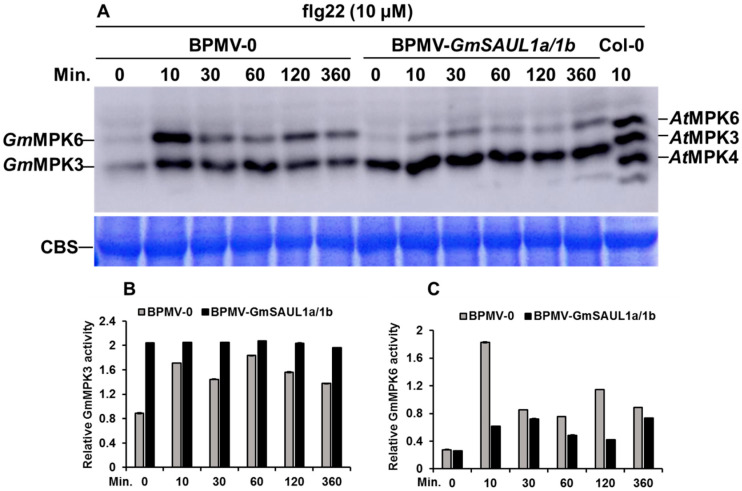
Silencing *GmSAUL1a/1b* reduces the activation of *Gm*MPK6 and enhances the activation *Gm*MPK3 in response to flg22 treatment. (**A**) Leaf discs from both BPMV-0 and BPMV-*SAUL1a/1b* plants were incubated on the moisture filter paper for 24 h to allow recovery from wounding before being treated with 10 μM flg22 or diluted DMSO over the indicated times. Activation of the kinase activities was detected by Western blotting using the Phosph-p44/p42 MAP Erk1/2 antibody. Arabidopsis sample treated with 10 μM flg22 for 10 min was used as a positive control for the purpose of band alignment. Coomassie Blue stained gel (CBS) was used as loading controls. (**B**) Relative *Gm*MPK3 activity shown in (**A**). (**C**) Relative *Gm*MPK6 activity shown in (**A**). Relative activity was calculated as the band intensities on the Western blot divided by the corresponding band intensities on the CBS gel.

The activated defense responses are usually associated with the downstream MAPK signaling pathway [28]. To examine the effect of the *GmSAUL1a/1b* silencing on the MAPK signaling activation, the kinase activity assay was performed for the leaf discs collected from both the BPMV-0 and the *GmSAUL1a/1b-*silenced plants treated with flg22, a 22 amino acid peptide at the N-terminus of the flagellin protein that is recognized by FLS2 [52], for a different period of time using Phospho-p44/42 MAP Erk1/2 antibody raised from human cells that can specifically recognize the phosphorylation of Arabidopsis MPK3/4/6 [53]. As shown in Figure 5, in response to flg22 elicitation, the activation of the *Gm*MPK6 was significantly reduced in the *GmSAUL1a/1b-*silenced plants relative to the BPMV-0 plants, whereas the activation of the *Gm*MPK3 was significantly elevated in the *GmSAUL1a/1b-*silenced plants, indicating that *Gm*SAUL1a/1b positively regulates the activation of the *Gm*MPK6, but negatively regulates the activation of *Gm*MPK3.

### 2.5. GmSAUL1a Is a Bona Fide E3 Ubiquitin Ligase

**Figure 6:**
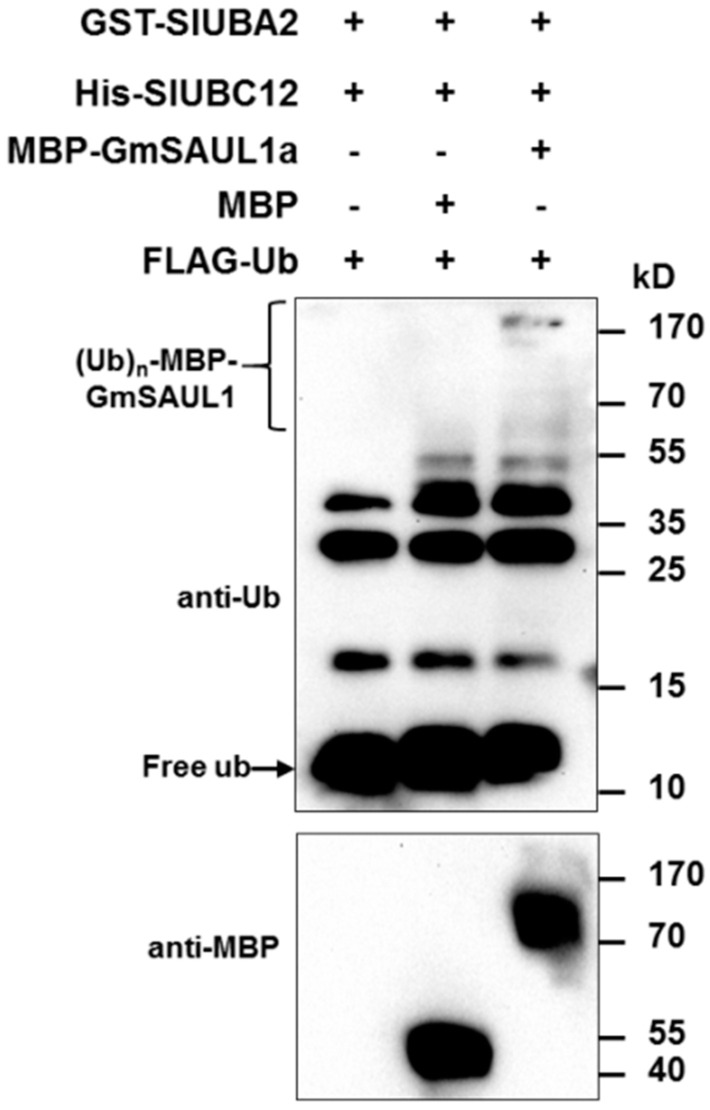
*Gm*SAUL1a is an active ubiquitin E3 ligase. The in vitro ubiquitin E3 ligase activity of *Gm*SAUL1a was performed in the presence of FLAG-ubiquitin (FLAG-Ub) as well as the recombinant E1 (GST-SlUBA2) and E2 (SlUBC12). Auto-polyubiquitination of MBP-*Gm*SAUL1a demonstrates its ubiquitin ligase activity. Anti-MBP and anti-Ub antibodies were used to detect MBP-*Gm*SAUL1a and free FLAG-Ub, respectively.

To examine whether *Gm*SAUL1a has ubiquitin E3 ligase activity, we incubated the *E. coli*-expressed recombinant protein MBP-*Gm*SAUL1a in the presence of ubiquitin, the E1 ubiquitin-activating enzyme SlUBA2, and the E2 ubiquitin-conjugating enzyme SlUBC12 [14]. As shown in Figure 6, a clear E3 ligase activity was detected for the MBP-*Gm*SAUL1a, indicating that *Gm*SAUL1a is a bona fide E3 ubiquitin ligase.

## 3. Discussion

### 3.1. Function of SAUL1 Homologs Is Conserved across Plant Species

In Arabidopsis, the loss function of SAUL1 leads to auto-immune phenotypes [20,21,23]. Here, we showed that silencing *GmSAUL1a/1b* in soybean resulted in similar autoimmune phenotypes (Figure 1, Figure 2 and Figure 3). The autoimmune phenotypes of the *saul1-1* mutant fully depend on PAD4 and EDS1 [20,21], suggesting that the autoimmune phenotypes of the *saul1-1* mutant are SA-dependent. Similarly, we found that both the free SA and conjugated SA levels were significantly higher in the *GmSAUL1a/1b*-silenced plants than in vector control plants (Figure 2C,D), implying that the autoimmunity in *GmSAUL1a/1b*-silenced plants is also SA-dependent. Consistent with the auto-immune phenotypes, the soybean *GmSAUL1a/1b*-silenced plants displayed an enhanced resistance to different types of biotrophic pathogens (Figure 3), indicating that the function of SAUL1 homologs is highly conserved between Arabidopsis and soybean.

### 3.2. Roles of GmSAUL1s in PTI and ETI

Silencing *GmSAUL1a/1b* in soybean resulted in enhanced resistance to virulent pathogens, which are considered as PTI (Figure 3). If the homeostasis of *Gm*SAUL1 is similarly guarded by NLR immune receptors such as SOC3-TN2 and SOC3-CHS1 pairs in Arabidopsis [23,24], then the activated immunity observed in the *GmSAUL1a/1b*-silenced soybean plants could actually be a consequence of activated NLRs. Higher temperature treatment significantly reversed the autoimmune phenotype observed in the *GmSAUL1a/1b-*silenced plants (Figure 4), suggesting that certain NLRs function as guards to monitor the homeostasis of *Gm*SAUL1s in soybean. Because PTI and ETI share common components and mutually potentiate each other to achieve stronger immunity [54,55], it is not surprising that the enhanced PTI observed in the *GmSAU1a/1b*-silenced soybean plants (Figure 3) could originate from the activated ETI. It is worthwhile to further examine whether the same pairs of SOC3-TN2 and SOC3-CHS1 homologs function to guard *Gm*SAUL1s in soybean.

### 3.3. Silencing of GmSAUL1a/1b in Soybean Activates Immunity through Activating GmMPK3

**Figure 7:**
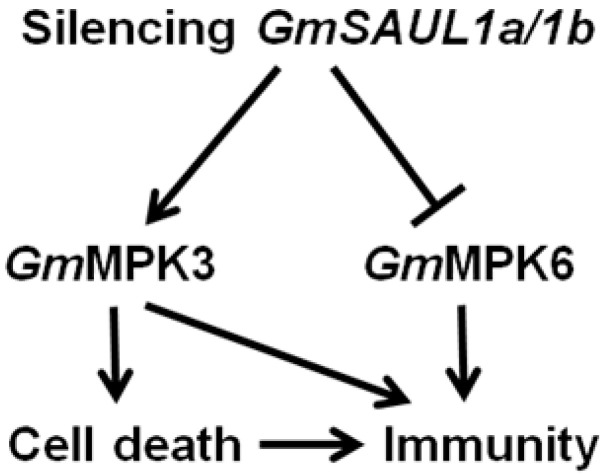
Silencing *GmSAUL1a/1b* triggers cell death through enhancing the activation of *Gm*MPK3. The elevated immunity in *GmSAUL1a/1b-*silenced plants could be the result of the cell death triggered by the activated *Gm*MPK3 as well as by the compensation effect of *Gm*MPK3 for the reduced activation of *Gm*MPK6.

Silencing *GmSAUL1a/1b* leads to the reduced activation of *Gm*MPK6, but the enhanced activation of *Gm*MPK3 in response to flg22 treatment (Figure 5), indicating that *Gm*SAUL1a/1b positively regulates *Gm*MPK6 activation and negatively regulates *Gm*MPK3. The enhanced resistance is usually correlated with the elevated activation of MPK3/MPK6 activity and reduced activation of MPK4, respectively [28,29,30,31,35,36,37,38]. We previously showed that the elevated activation of *Gm*MPK3 was associated with the cell death observed in the *GmMPK4*-, *GmMPK6*-, and *GmMEKK1*-silenced plants [45], which is consistent with the finding that the enhanced activation of MPK3 resulted in cell death [56]. Therefore, it is likely that the cell death that occurred on the leaves of the *GmSAUL1a/1b*-silenced plants was a result of the enhanced activation of *Gm*MPK3. It has been reported that, in Arabidopsis, the activation MPK6 is elevated in loss-of-function mutants of *MPK3* and vice versa [57], suggesting that MPK3 and MPK6 can mutually compensate for each other’s function. If this holds true in soybean, the elevated activation of *Gm*MPK3 might compensate for the reduced activation of *Gm*MPK6 in *GmSAUL11a/1b*-silenced plants (Figure 5), which is responsible for the enhanced disease resistance observed in the *GmSAUL1a/1b*-silenced plants (Figure 3). Collectively, our results indicated that silencing *GmSAUL1a/1b* activates immune responses through activating *Gm*MPK3 (Figure 7). It remains to be determined whether the activated immunity in the *GmSAUL1a/1b*-silenced plants is a result of the activation of an NLR.

### 3.4. Conclusions

Using the BPMV-VIGS system in soybean, we demonstrated that *Gm*SAUL1a/1b plays a negative role in in soybean immunity. The fact that the activated immune responses could be rescued by high temperature treatment suggests that the activated immunity observed in the silenced plant is probably guarded by one or more NLR proteins. Unexpectedly, we found that silencing *GmSAUL1a/1b* resulted in the activation of *Gm*MPK3, but the repression of *Gm*MPK6. Most importantly, we showed that *Gm*SAUL1a is a bona fide U-box E3 ligase. In sum, our results indicated that *Gm*SAUL1a/1b plays a negative role in regulating immunity, likely through repressing the activation of *Gm*MPK3.

## 4. Materials and Methods

### 4.1. Plant Materials

Seeds of soybean (*Glycine max* ‘Williams 82) were provided by Prof. Steven Whitham at Iowa State University and used in this study. Soybean plants were maintained in the growth room or growth chamber at 22 °C with a photoperiod of 16 h light/8 h dark, unless indicated otherwise.

### 4.2. BPMV-Mediated VIGS

The BPMV-VIGS system and its usage have been described previously [58,59]. The *GmSAUL1* orthologs were identified by BLASTn searches (cutoff value < 0.001) in the Phytozome database on 6 October 2019 (www.phytozome.org). A 360 bp fragment of *GmSAUL1a* was amplified by PCR using the following primers: *GmSAUL1a*-F (*Glyma*.04G016500), 5′-aag**GGATCC**GTTATGCGTGATCCTGTTACTTTAGA-3′, and *GmSAUL1a*-R: 5′-ttg**GGTACC**CCTTCAGCATGTCAACAATCATTG-3′. The bold sequences represent BamHI and KpnI restriction sites for the cloning purpose, respectively.

### 4.3. RNA Isolation and RT-qPCR

RNA isolation and RT-qPCR were performed as described [40]. The RT-qPCR tests were performed using an ABI550 Real-Time PCR machine (Applied Biosystems, Thermo Fisher Scientific, Austin, TX, USA) and the 2x SYBR Green qPCR Mix (Aidlab, Beijing, China). The primers used for verification of the effects of silencing of *GmSAUL1a/1b*, were:

*GmSAUL1a/1b*-V-F: ATGATGGCTGCGAGCT;

*GmSAUL1a/1b*-V-R: CTCATGTGAAAGGAATTTTACTAC.

The additional primers used in this study were:

*GmSAUL1a/1b*-F: ATGATGGCTGCGAGCT;

*GmSAUL1a/1b*-R: CTCATGTGAAAGGAATTTTACTAC;

*GmELF1b-F*: ACCGAAGAGGGCATCAAATCCC;

*GmELF1b-R:* CTCAACTGTCAAGCGTTCCTC;

*GmELF1b-F* (RT-qPCR): GTTGAAAAGCCAGGGGACA;

*GmELF1b-R* (RT-qPCR): TCTTACCCCTTGAGCGTGG;

*GmPR1-F* (RT-qPCR): ATGGGGTTGTGCAAGGTT;

*GmPR1-R* (RT-qPCR): CTAGTAGGGTCTTTGGCCAA.

### 4.4. SMV-N-GUS Inoculation, GUS Staining, and GUS Foci Measurements

The SMV-N-GUS inoculation, GUS staining, and GUS foci measurements followed the previously described protocols [39,48,60].

### 4.5. Inoculation of Pseudomonas syringae pv. glycinea (Psg)

The *Psg* inoculation and growth assay was performed as described [46].

### 4.6. Construction of MBP-GmSAUL1a Fusion Protein and In Vitro Ubiquitination Assay

The full-length cDNA of *GmSAUL1a* was cloned into pMAL-c2 vector (New England Biolabs, Ipswich, MA, USA). The primers used for the making the construct were:

pMAL-SAUL1 -BamH I-F:

aaaGGATCCATGATGGCTGCGAGCT.

pMAL-MBP-HinD III-R:

tttAAGCTTTCATCCCATGTTTGGAAAGATTC.

The construct was transformed into *E. coli* strain BL21 Star (DE3) (Invitrogen) and protein expression and purification were performed as described previously [61]. The in vitro ubiquitination assay was carried out as described previously with some modifications [16,17]. Briefly, 3 μg of ubiquitin, 40 ng of E1 (GST- SlUBA2), an optimal amount (50–250 ng) of E2 (6xHis-SlUBC12), and 2 μg of MBP-*Gm*SAUL1 were added to a 30 mL reaction in the presence of ubiquitination assay buffer (50 mM Tris-HCl, pH 7.5, 5 mM ATP, 5 mM MgCl_2_, 2 mM DTT, 3 mM creatine phosphate, and 5 μg/mL creatine phosphokinase). The reactions were incubated at 30 °C for 1.5 h and then terminated by SDS sample loading buffer with 100 mM DTT. The samples were heated at 95 °C for 5 min and then separated using 10% SDS-PAGE and analyzed by immunoblotting using mouse monoclonal anti-ubiquitin M2-peroxidase-conjugated (horseradish peroxidase, HRP) antibody (Santa Cruz Biotechnology, Santa Cruz, CA, USA) to detect the polyubiquitin signal. The MBP and polyubiquitinated form of MBP-*Gm*SAUL1 were detected using murine anti-MBP monoclonal antibody (HRP conjugated) (NEB).

### 4.7. MAPK Activity Assay

MAPK activity assay was performed as described [45].

### 4.8. Histochemical Assays

The visualization of cell death on the leaves the soybean plants were stained with trypan blue in lactophenol and ethanol as described [62]. The accumulation of H_2_O_2_ was stained using the 3,3-diaminobenzidine tetrahydrochloride (DAB) staining procedure (Sigma-Aldrich; [62]).

### 4.9. SA Quantification

SA was quantified using an Agilent 1260 HPLC system (Agilent Technologies, Santa Clara, CA, USA) with a diode array detector, a fluorescence detector, and a column, as described previously [63].

## Data Availability

Not applicable.

## References

[1] Schulze-Lefert P., Panstruga R. (2011). A molecular evolutionary concept connecting nonhost resistance, pathogen host range, and pathogen speciation. Trends Plant Sci..

[2] Jones J.D.G., Dangl J.L. (2006). The plant immune system. Nature.

[3] Chinchilla D., Zipfel C., Robatzek S., Kemmerling B., Nürnberger T., Jones J.D.G., Felix G., Boller T. (2007). A flagellin-induced complex of the receptor FLS2 and BAK1 initiates plant defence. Nature.

[4] Boller T., Felix G. (2009). A renaissance of elicitors: Perception of microbe-associated molecular patterns and danger signals by pattern-recognition receptors. Annu. Rev. Plant Biol..

[5] Yuan M., Ngou B.P.M., Ding P., Xin X.F. (2021). PTI-ETI cross-talk: An integrative view of plant immunity. Curr. Opin. Plant Biol..

[6] Tena G., Boudsocq M., Sheen J. (2011). Protein kinase signaling networks in plant innate immunity. Curr. Opin. Plant Biol..

[7] Zhou J., Zhang Y. (2020). Plant Immunity: Danger Perception and Signaling. Cell.

[8] Vierstra R.D. (2009). The ubiquitin-26S proteasome system at the nexus of plant biology. Nat. Rev. Mol. Cell Biol..

[9] Zhang Y., Zeng L. (2020). Crosstalk between ubiquitination and other post-translational protein modifications in plant immunity. Plant Commun..

[10] Smalle J., Viestra R.D. (2004). The Ubiquitin 26S proteasome proteolytic pathway. Annu. Rev. Plant Biol..

[11] Hershko A., Clechanover A. (1998). The ubiquitin system. Annu. Rev. Biochem..

[12] Cheng Y.T., Li X. (2012). Ubiquitination in NB-LRR-mediated immunity. Curr. Opin. Plant Biol..

[13] Zeng L., Vega-Sanchez M.E., Zhu T., Wang G.L. (2006). Ubiquitination-mediated protein degradation and modification: An emerging theme in plant-microbe interactions. Cell Res..

[14] Zhou B., Zeng L. (2017). Conventional and unconventional ubiquitinnation in plant immunity. Mol. Plant Pathol..

[15] Santner A., Estelle M. (2010). The ubiquitin-proteasome system regulates plant hormone signaling. Plant J..

[16] Zeng L., Qu S., Bordeos A., Yang C., Baraoidan M., Yan H., Xie Q., Nahm B.H., Leung H., Wang G.L. (2004). Soptted leaf 11, a negative regulator of plant cell death and defense, encodes a U-box/armadillo repeat protein endowed with E3 ubiquitin ligase activity. Plant Cell.

[17] Lu D., Lin W., Gao X., Wu S., Cheng C., Avila J., Heese A., Devarenne T.P., He P., Shan L. (2011). Direct ubiquitination of pattern recognition receptor FLS2 attenuates plant innate immunity. Science.

[18] Stegmann M., Anderson R.G., Ichimura K., Pecenkova T., Reuter P., Žársky V., McDowell J.M., Shirasu K., Trujillo M. (2012). The ubiquitin ligase PUB22 targets a subunit of the exocyst complex required for PAMP-triggered responses in Arabidopsis. Plant Cell.

[19] Raab S., Drechsel G., Zarepour M., Hartung W., Koshiba T., Bittner F., Hoth S. (2009). Identification of a novel E3 ubiquitin ligase that is required for suppression of premature senescence in Arabidopsis. Plant J..

[20] Vogelmann K., Drechsel G., Bergler J., Subert C., Philippar K., Soll J., Engelmann J.C., Engelsdorf T., Voll L.M., Hoth S. (2012). Early senescence and cell death in Arabidopsis saul1 mutants involves the PAD4-dependent salicylic acid pathway. Plant Physiol..

[21] Disch E.M., Tong M., Kotur T., Koch G., Wolf C.A., Li X., Hoth S. (2016). Membrane-associated ubiquitin ligase SAUL1 suppresses temperature-and humidity-dependent autoimmunity in Arabidopsis. Mol. Plant-Microbe Interact..

[22] Lee I.H., Lee I.C., Kim J., Kim J.H., Chung E.H., Kim H.J., Park S.J., Kim Y.M., Kang S.K., Nam H.G. (2016). NORE1/SAUL1 integrates temperature-dependent defense programs involving SGT1b and PAD4 pathways and leaf senescence in Arabidopsis. Physio. Plant..

[23] Tong M., Kotur T., Liang W., Vogelmann K., Kleine T., Leister D., Brieske C., Yang S., Ludke D., Wiermer M. (2017). E3 ligase SAUL1 serves as a positive regulator of PAMP-triggered immunity and its homeostasis is monitored by immune receptor SOC3. New Phytol..

[24] Liang W., van Wersch S., Tong M., Li X. (2019). TIR-NB-LRR immune receptor SOC3 pairs with truncated TIR-NB protein CHS1 or TN2 to monitor the homeostasis of E3 ligase SAUL1. New Phytol..

[25] Zhang Y., Wang Y., Liu J., Ding Y., Wang S., Zhang X., Liu Y., Yang S. (2016). Temperature-dependent autoimmunity mediated by *chs1* requires its neighboring TNL gene SOC3. New Phytol..

[26] Colcombet J., Hirt H. (2008). Arabidopsis MAPKs: A complex signal-ling network involved in multiple biological processes. Biochem. J..

[27] Pitzschke A., Schikora A., Hirt H. (2009). MAPK cascade signalling networks in plant defence. Curr. Opin. Plant Biol..

[28] Meng X., Zhang S. (2013). MAPK cascades in plant disease resistance signaling. Annu. Rev. Phytopathol..

[29] Zhang S., Klessig D.F. (1998). Resistance gene *N*-mediated *de novo* synthesis and activation of a tobacco mitogen-activated protein kinase by *Tobacco mosaic virus* infection. Proc. Natl. Acad. Sci. USA.

[30] Zhang S., Liu Y. (2001). Activation of salicylic acid-induced protein kinase, a mitogen-activated protein kinase, induces multiple defense responses in tobacco. Plant Cell.

[31] Menke F.L., van Pelt J.A., Pieterse C.M., Klessig D.F. (2004). Silencing of the mitogen-activated protein kinase MPK6 compromises disease resistance in Arabidopsis. Plant Cell.

[32] Mao G., Meng X., Liu Y., Zheng Z., Chen Z., Zhang S. (2011). Phosphorylation of a WRKY transcription factor by two pathogen-responsive MAPKs drives phytoalexin biosynthesis in Arabidopsis. Plant Cell.

[33] Ren D., Liu Y., Yang K.Y., Han L., Mao G., Glazebrook J., Zhang S. (2008). A fungal-responsive MAPK cascade regulates phytoalexin biosynthesis in Arabidopsis. Proc. Natl. Acad. Sci. USA.

[34] Petersen M., Brodersen P., Naested H., Andreasson E., Lindhart U., Johansen B., Nielsen H.B., Lacy M., Austin M.J., Parker J.E. (2000). Arabidopsis MAP Kinase 4 Negatively Regulates Sy Liang, W.stemic Acquired Resistance. Cell.

[35] Qiu J.L., Zhou L., Yun B.W., Nielsen H.B., Fiil B.K., Petersen K., Mackinlay J., Loake G.J., Mundy J., Morris P.C. (2008). Arabidopsis mitogen-activated protein kinase kinases MKK1 and MKK2 have overlapping functions in defense signaling mediated by MEKK1, MPK4, and MKS1. Plant Physiol..

[36] Gao M., Liu J., Bi D., Zhang Z., Cheng F., Chen S., Zhang Y. (2008). MEKK1, MKK1/MKK2 and MPK4 function together in a mitogen-activated protein kinase cascade to regulate innate immunity in plants. Cell Res..

[37] Gao M., Liu J., Bi D., Zhang Z., Cheng F., Chen S., Zhang Y. (2012). The MEKK1-MKK1/MKK2-MPK4 kinase cascade negatively regulates immunity mediated by a mitogen-activated protein kinase kinase kinase in Arabidopsis. Plant Cell.

[38] Pitzschke A., Djamei A., Bitton F., Hirt H. (2009). A major role of the MEKK1-MKK1/2-MPK4 pathway in ROS signalling. Mol. Plant.

[39] Liu J.Z., Horstman H.D., Braun E., Graham M.A., Zhang C., Navarre D., Qiu W.L., Lee Y., Nettleton D., Hill J.H. (2001). Soybean homologs of MPK4 negatively regulate defense responses and positively regulate growth and development. Plant Physiol..

[40] Liu J.Z., Braun E., Qiu W.L., Shi Y.F., Marcelino-Guimars F.C., Navarre D., Hill J.H., Whitham S.A. (2014). Positive and negative roles for soybean MPK6 in regulating defense responses. Mol. Plant Microbe Interact..

[41] Liu J.Z., Graham M.A., Pedley K.F., Whitham S.A. (2015). Gaining insight into soybean defense responses using functional genomics approaches. Brief. Funct. Genom..

[42] Liu J.Z., Fang Y., Pang H. (2016). The current status of the soybean-soybean mosaic virus (SMV) pathosystem. Front. Microbiol..

[43] Schmutz J., Cannon S.B., Schlueter J., Ma J., Mitros T., Nelson W., Hyten D.L., Song Q., Thelen J.J., Cheng J. (2010). Genome sequence of the palaeopolyploid soybean. Nature.

[44] Liu D.D., Lan H.J., Masoud H.S., Ye M.Y., Dai X.Y., Zhong C.L., Tian S.N., Liu J.Z. (2022). Silencing *GmBIR1* in Soybean Results in Activated Defense Responses. Int. J. Mol. Sci..

[45] Xu H.Y., Zhang C., Li Z.C., Wang Z.R., Jiang X.X., Shi Y.F., Tian S.N., Braun E., Mei Y., Qiu W.L. (2018). The MAPK kinase kinase GmMEKK1 regulates cell death and defense responses. Plant Physiol..

[46] Tian S.N., Liu D.D., Zhong C.L., Xu H.Y., Yang S., Fang Y., Ran J., Liu J.Z. (2020). Silencing *GmFLS2* enhances the susceptibility of soybean to bacterial pathogen through attenuating the activation of *Gm*MAPK signaling pathway. Plant Sci..

[47] Liu J.Z., Whitham S.A. (2013). Over-expression of a nuclear-localized DnaJ domain-containing HSP40 from soybean reveals its roles in cell death and disease resistance. Plant J..

[48] Wang L., Eggenberger A., Hill J., Bogdanove A.J. (2006). *Pseudomonas syringae* effector avrB confers soybean cultivar-specific avirulence on Soybean mosaic virus adapted for transgene expression but effector avrPto does not. Mol. Plant Microbe Interact..

[49] Whitham S., Dinesh-Kumar S.P., Choi D., Hehl R., Corr C., Baker B. (1994). The product of the tobacco mosaic virus resistance gene N: Similarity to toll and the interleukin-1 receptor. Cell.

[50] Whitham S., McCormick S., Baker B. (1996). The N gene of tobacco confers resistance to tobacco mosaic virus in transgenic tomato. Proc. Natl. Acad. Sci. USA.

[51] Zhu Y., Qian W., Hua J. (2010). Temperature Modulates Plant Defense Responses through NB-LRR Proteins. PLoS Pathog..

[52] Zipfel C., Robatzek S., Navarro L., Oakeley E.J., Jones J.D.G., Felix G., Boller T. (2004). Bacterial disease resistance in Arabidopsis through flagellin perception. Nature.

[53] Zhao C., Nie H., Shen Q., Zhang S., Lukowitz W., Tang D. (2014). EDR1 physically interacts with MKK4/MKK5 and negatively regulates a MAP kinase cascade to modulate plant innate immunity. PLoS Genet..

[54] Yuan M., Jiang Z., Bi G. (2021). Pattern-recognition receptors are required for NLR-mediated plant immunity. Nature.

[55] Ngou B.P.M., Ahn H.K., Ding P., Jones J. (2021). Mutual potentiation of plant immunity by cell-surface and intracellular receptors. Nature.

[56] Genot B., Lang J., Berriri S., Garmier M., Gilard F., Pateyron S., Haustraete K., Van Der Straeten D., Hirt H., Colcombet J. (2017). Constitutively active Arabidopsis MAP Kinase 3 triggers defense responses involving salicylic acid and SUMM2 resistance protein. Plant Physiol..

[57] Wang H., Liu Y., Bruffett K., Lee J., Hause G., Walker J., Zhang S. (2008). Haplo-insufficiency of *MPK3* in *MPK6* mutant background uncovers a novel function of these two MAPKs in Arabidopsis ovule development. Plant Cell.

[58] Zhang C., Yang C., Whitham S.A., Hill J.H. (2009). Development and use of an efficient DNA-based viral gene silencing vector for soybean. Mol. Plant Microbe Interact..

[59] Zhang C., Bradshaw J.D., Whitham S.A., Hill J.H. (2010). The development of an efficient multipurpose bean pod mottle virus viral vector set for foreign gene expression and RNA silencing. Plant Physiol..

[60] Jefferson R.A., Kavanagh T.A., Bevan M.W. (1987). GUS fusions: Beta-glucuronidase as a sensitive and versatile gene fusion marker in higher plants. EMBO J..

[61] Liu J.Z., Duan J., Whitham S.A., Qian W.J. (2017). S-nitrosylation inhibits the kinase activity of tomato phosphoinositide-dependent kinase 1 (PDK1). J. Biol. Chem..

[62] Ren D., Yang H., Zhang S. (2002). Cell death mediated by MAPK is associated with hydrogen peroxide production in Arabidopsis. J. Biol. Chem..

[63] Zhang Y., Zhao L., Zhao J., Li Y., Wang J., Guo R., Gan S., Liu C.J., Zhang K. (2017). *S5H/DMR6* Encodes a Salicylic Acid 5-Hydroxylase That Fine-Tunes Salicylic Acid Homeostasis. Plant Physiol..

